# Towards the Investigation of the Adaptive Divergence in a Species of Exceptional Ecological Plasticity: Chromosome-Scale Genome Assembly of *Chouardia litardierei* (Hyacinthaceae)

**DOI:** 10.3390/ijms241310755

**Published:** 2023-06-28

**Authors:** Ivan Radosavljević, Krešimir Križanović, Sara Laura Šarančić, Jernej Jakše

**Affiliations:** 1 Division of Botany, Department of Biology, Faculty of Science, University of Zagreb, Marulićev trg 9A, HR-10000 Zagreb, Croatia; 2 Department of Electronic Systems and Information Processing, Faculty of Electrical Engineering and Computing, University of Zagreb, Unska 3, HR-10000 Zagreb, Croatia; 3 Department of Agronomy, Biotechnical Faculty, University of Ljubljana, Jamnikarjeva 101, SI-1000 Ljubljana, Slovenia

**Keywords:** *Chouardia litardierei*, PacBio, Hi-C, chromosome-level genome, draft genome, local adaptation

## Abstract

One of the central goals of evolutionary biology is to understand the genomic basis of adaptive divergence. Different aspects of evolutionary processes should be studied through genome-wide approaches, therefore maximizing the investigated genomic space. However, in-depth genome-scale analyses often are restricted to a model or economically important species and their closely related wild congeners with available reference genomes. Here, we present the high-quality chromosome-level genome assembly of *Chouardia litardierei*, a plant species with exceptional ecological plasticity. By combining PacBio and Hi-C sequencing technologies, we generated a 3.7 Gbp genome with a scaffold N50 size of 210 Mbp. Over 80% of the genome comprised repetitive elements, among which the LTR retrotransposons prevailed. Approximately 86% of the 27,257 predicted genes were functionally annotated using public databases. For the comparative analysis of different ecotypes’ genomes, the whole-genome sequencing of two individuals, each from a distinct ecotype, was performed. The detected above-average SNP density within coding regions suggests increased adaptive divergence-related mutation rates, therefore confirming the assumed divergence processes within the group. The constructed genome presents an invaluable resource for future research activities oriented toward the investigation of the genetics underlying the adaptive divergence that is likely unfolding among the studied species’ ecotypes.

## 1. Introduction

**Figure 1:**
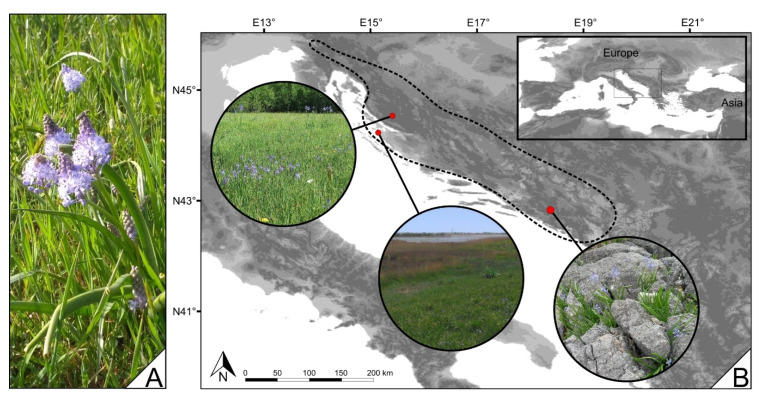
(**A**) *Chouardia litardierei* in full bloom, (**B**) the distribution area of *Chouardia litardierei* and contrasting habitat types it occupies. The distribution area of *Chouardia litardierei* is marked with a dotted line. In circles, from left to right, meadow, seashore, and mountainous ecotype habitats are shown.

Amethyst meadow squill (*Chouardia litardierei* (Breist.) Speta) (Figure 1A) is a bulbous perennial species of the Hyacinthaceae family. It grows naturally across the western and central parts of the Dinaric Alps in the Balkan Peninsula, occupying highly contrasting ecological niches [1,2] and therefore meadow, seashore, and mountainous ecotypes can be recognized (Figure 1B).

The meadow ecotype, distributed throughout the central and northern parts of the species distribution area, is found across karst fields at altitudes of up to 1000 m. These flat-floored and periodically flooded enclosed depressions are characterized by a unique microclimate and hydrological and geomorphological conditions compared to the surrounding areas [3]. The seashore ecotype occupies the lowlands of northern Dalmatia across the northwestern part of the species distribution range. These populations grow in salt marshes reaching the seashore, which experience Mediterranean climate conditions [4,5]. Finally, the mountainous ecotype is distributed throughout the southern parts of the species’ distribution range and in comparison to the aforementioned two ecotypes, occupies a highly contrasting habitat. Its populations inhabit arid, rocky slopes of high mountains with very little or virtually no soil in rock crevices at altitudes of up to 2000 m that are characterized by extreme seasonality of most climatic elements. Despite occupying contrasting environments, these groups of populations can hardly be distinguished from each other by any morphological trait. There was an attempt to describe the mountainous ecotype as a separate taxon based on morphological and phenological analyses [2], but the research was based on vague and unreliable approaches, therefore leaving room for justified doubts in the results. *C. litardierei* undoubtedly is a complex species characterized by very pronounced ecological plasticity. However, unlike in some other cases [6], it seems only the specific habitat, and not any morphological trait, can be used for reliable recognition of the ecotypes. We plan to use this species as a study system for a thorough investigation of the genetics underlying the ecological divergence and speciation process.

To date, no significant research that investigated this species’ ecological divergence or genetics has been performed. Besides the previously mentioned analyses by Šilić [2], the cytogenetic characterization of two individuals representing meadow and mountainous ecotypes was also performed [7]. Karyograms revealed that both ecotypes share the same number of chromosomes (2n = 26), with one long, two middle-sized, and ten small chromosome pairs. In addition, the 1C haploid genome size was estimated at 4.13 pg [8] or 4.039 Gbp according to the conversion by Doležel et al. [9].

During the process of speciation, a group of individuals diverges into two or more distinct phylogenetic lineages. In populations initially indistinguishable from each other, either genetically or morphologically, the accumulation of genetic differences can gradually lead to the emergence of a new species [10,11]. The type of speciation in which “barriers to gene flow evolve between populations as a result of ecologically based divergent selection” is referred to as ecological speciation [12]. As a consequence of organism adaptation to specific environmental conditions during ecological speciation, new morphologically and genetically divergent ecotypes found in a specific habitat rather than a specific geographic area, can emerge [13]. One of the central goals of evolutionary biology is to understand the genomic basis of adaptive evolution [14,15]. It is widely accepted that different aspects of evolutionary processes should be studied through genome-wide approaches, therefore maximizing the investigated genomic space. However, genome-scale analyses are often restricted to a model or economically important species (and their closely related wild congeners) with available high-quality reference genomes [16,17,18]. In recent years, with the advancement of different NGS techniques and the inevitable increase in their affordability, more non-model species’ genomes are being sequenced and assembled de novo [19,20,21].

Here, we present the high-quality chromosome-scale genome assembly for *C. litardierei*, which is also, to the best of our knowledge, the first reported genome assembly within the Hyacinthaceae family. By implementing PacBio HiFi sequencing and Hi-C scaffolding, a haploid 3.7 Gb genome organized in 13 pseudochromosomes was revealed. The obtained results represent the initial step in comprehensive research that will investigate the process of adaptive divergence and speciation that is likely unfolding among the ecotypes of the studied species. The availability of the species’ genome assembly will enable the study of the ecotypes’ genome architecture, genome–environment association (GEA), and genome-wide association studies (GWAS), which will elucidate the genomic mechanisms underlying the ongoing evolutionary processes in *C. litardierei*.

## 2. Results

### 2.1. Genome Sequencing and Assembly

After sequencing, high-quality PacBio CCS reads were obtained from subreads with a quality score of Q20 (1% error rate). More than 6.5 M PacBio HiFi reads were available with a total of 94.54 Gbp (23× genome coverage, genome size based on the *k*-mer analysis), producing an average read length of 14.5 Kbp. In addition, 861 M Hi-C read-pairs were obtained, resulting in 432 Gbp (105× genome coverage) in total. Based on *k*-mer analysis, the genome size of amethyst meadow squill was estimated at 4.085 Gbp. After processing the hifiasm assembly using Quast, the initial genome assembly of 3.67 Gbp with an average contig N50 of 12.9 Mbp was produced.

**Figure 2:**
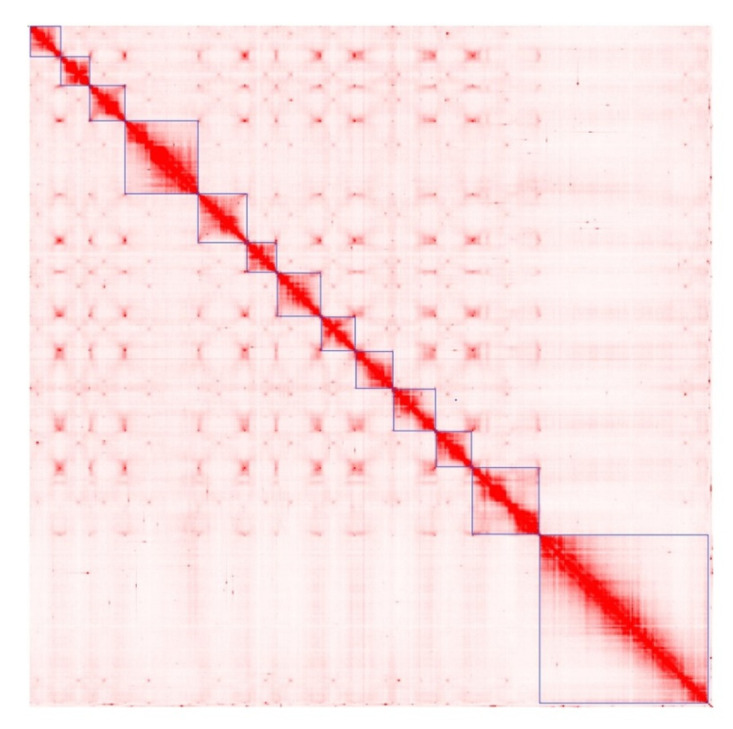
Heatmap showing Hi-C interactions for 13 pseudochromosomes of *Chouardia litardierei*.

**Figure 3:**
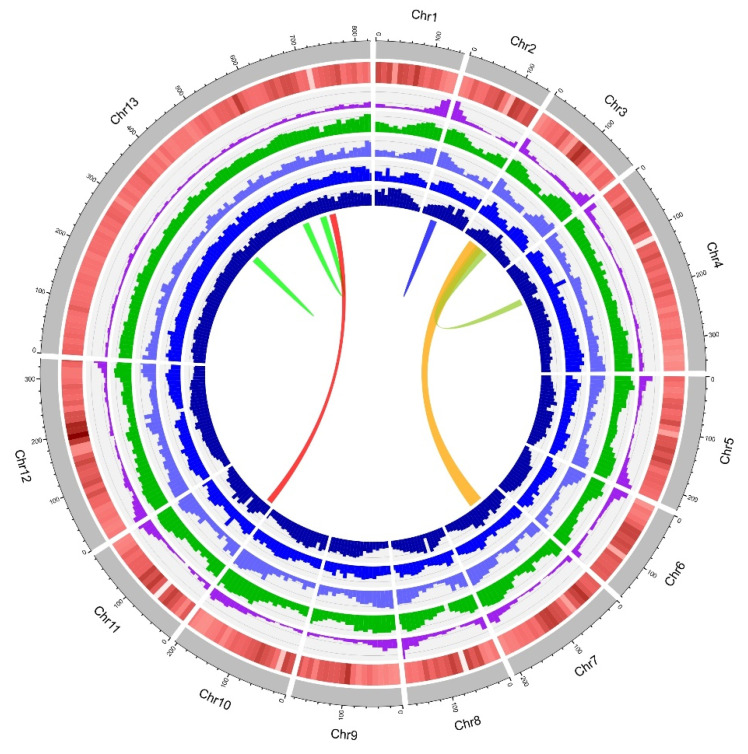
Genome features of 10 Mbp windows across the *Chouardia litardierei* genome. From outer to inner circles: chromosomes, GC content, gene density (purple), total repeats (green), DNA transposons density (light blue), *Copia* elements density (blue), *Gypsy* elements density (dark blue), and intra-genome syntenic blocks where the bandwidth is proportional to the syntenic block size.

After processing the initial assembly and Hi-C data with 3D-DNA, the assembly results were moderately improved and the scaffold N50 measure topped 200 Mbp. The N50 measure obtained after the 3D-DNA pipeline should be considered reliable due to misjoins having been resolved by the pipeline. The rearrangement of scaffolds produced by the 3D-DNA pipeline with the Juicebox tool resulted in the recognition of 13 pseudochromosomes: one very long, two middle-sized, and ten small chromosomes (Figure 2 and Figure 3). 

The obtained assembly was polished using the HyPo tool, and the results are presented in Table 1. The N50 value reached more than 210 Mbp, and the largest scaffold was nearly 825 Mbp. The 13 largest scaffolds (representing pseudochromosomes) range from 146 Mbp to 825 Mbp, with a total size of 3.33 Gbp. This value represents 90% of the complete assembly and 81.6% of the predicted genome length. The rest of the assembly consists of numerous smaller sequences (2.3 Mbp and smaller) that did not successfully merge with the pseudochromosomes. Finally, the BUSCO completeness score of 97.4% confirmed the high quality of the obtained genome assembly. The summary statistics are presented in Table 1.

### 2.2. Repetitive Elements Annotation

The annotation of repetitive elements revealed 2.99 Gbp of repetitive sequences representing 80.90% of the *C. litardierei* genome, with transposable elements (TEs) occupying 69.97% of the genome assembly. In addition, the analysis revealed that LTR retrotransposons were by far the most abundant repeat sequences (63.25% of the genome assembly), of which *Copia* and *Gypsy*, two superfamilies, account for 27.03% and 36.01% of the assembled sequences, respectively. Other detected repeat elements were unclassified elements (7.81%), DNA transposons (3.67%), long interspersed nuclear elements (LINEs; 2.99%), and others with lower abundances (Table 2).

### 2.3. RNA Sequencing

The RNA sequencing yielded a total of 99.59 M raw reads. After trimming, 96.75 M reads with an average length of 135.6 bp were retained. The summary of the RNA sequencing results from different tissues is given in Table 3.

### 2.4. Gene Prediction and Annotation

**Figure 4:**
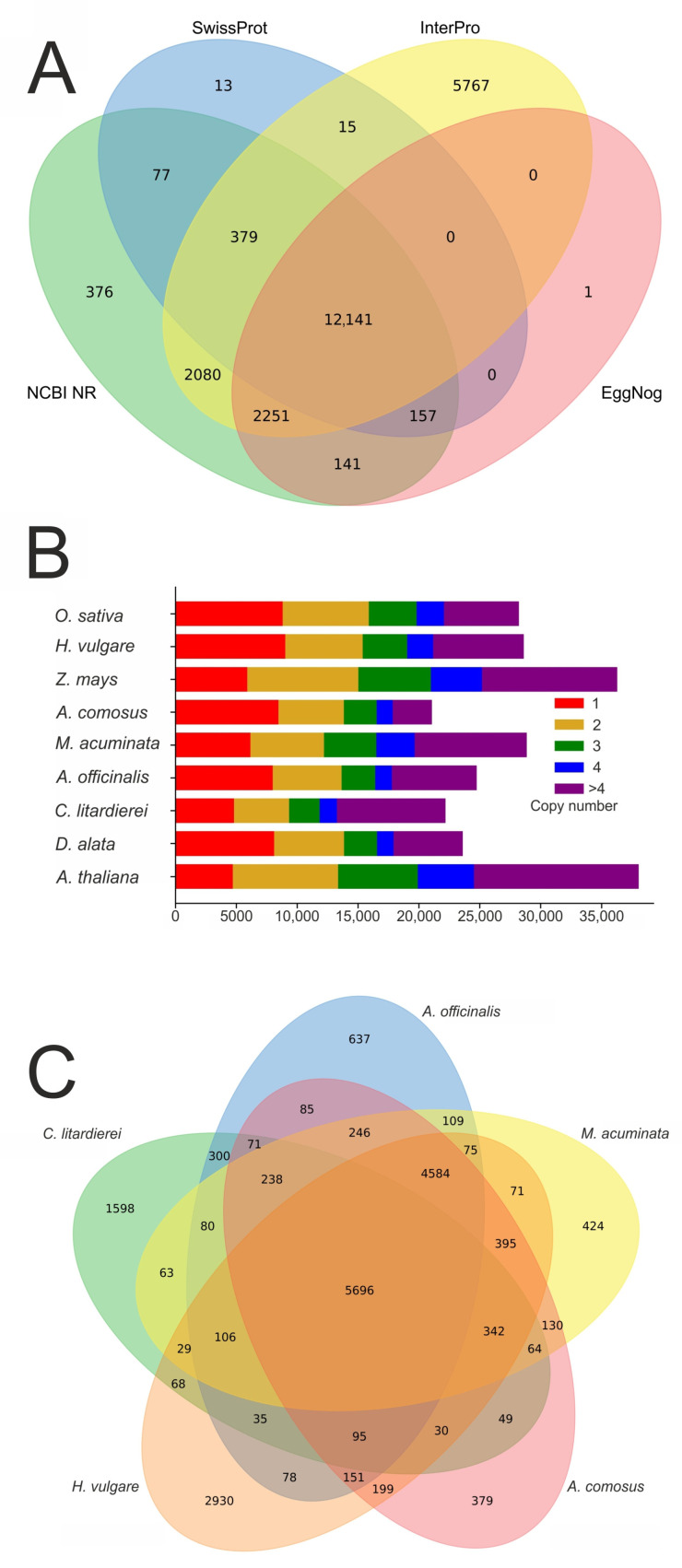
(**A**) Venn diagram showing the number of genes with functional annotation using multiple public databases, (**B**) number of gene copies among nine studied plant species, (**C**) Venn diagram of orthologous groups shared among selected species.

By combining several approaches, we predicted 27,257 gene models, of which 23,297 were mapped to 13 pseudochromosomes, while the remaining 3960 were mapped to smaller scaffolds. Their average length, CDS length, and exon number were 3109.9 bp, 764.1 bp, and 4.2 bp, respectively (Table 4). Among the predicted genes, 23,398 were functionally annotated using the public databases Swiss-Prot, InterPro, NCBI NR, and EggNog (Figure 4A). 

### 2.5. Evolution Analysis

**Figure 5:**
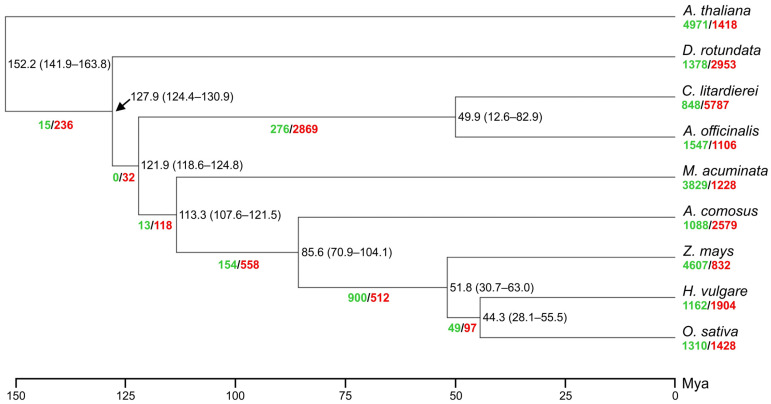
A phylogenetic tree showing topology, divergence times, and expansions/contractions of gene families for nine plant species including *Chouardia litardierei*. Numbers in green, red, and black represent expansions and contractions of gene families, and divergence times, respectively.

To elucidate the evolutionary history of *C. litardierei* within monocots, seven species across the group and one dicot (*A. thaliana* as an outgroup) were selected for the phylogenetic analysis. A total of 24,356 orthologous families of genes were identified: 377 single-copy families, 5189 shared by all studied species, 5486 shared only by monocots representatives, and 6621 shared by *C. litardierei* and *A. officinalis* (Figure 4C). For *C. litardierei* 1458 private gene families were recognized. Single-copy ortho-groups were used for the phylogenetic tree construction. Species formed groups that were in accordance with their already recognized phylogenetic relationships. *C. litardierei* paired with *A. officinalis* within the order Asparagales, while *Z. mays*, *H. vulgare*, and *O. sativa* grouped as representatives of the Poaceae family. As representatives of different families, *D. rotundata, M. acuminata*, and *A. comosus* were positioned separately, as was the case with *A. thaliana* as the sole representative of dicots that served as the outgroup. The divergence time between *C. litardierei* and *A. officinalis* was estimated at 49.9 Mya. The divergence times among the other analyzed species and gene family expansions and contractions are indicated in Figure 5. 

### 2.6. Ecotypes Genomes Comparison

**Figure 6:**
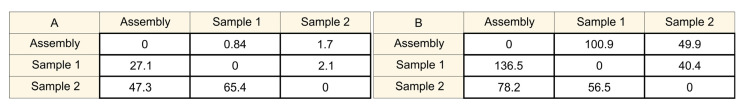
Genetic distances and distribution of SNPs among studied *Chouardia litardierei* genomes. Assembly—draft genome assembly of an individual belonging to the seashore ecotype; Sample 1—individual belonging to the meadow ecotype; Sample 2—individual belonging to the mountainous ecotype. (**A**) The total number of SNPs for the given sample pair is shown below the diagonal, and the number of SNPs detected in genes is shown above the diagonal (in millions). (**B**) Mean distance between neighboring SNPs throughout the genome for the given sample pair is shown below the diagonal, and the mean distance between neighboring SNPs within detected genes is shown above the diagonal (in base pairs).

**Figure 7:**
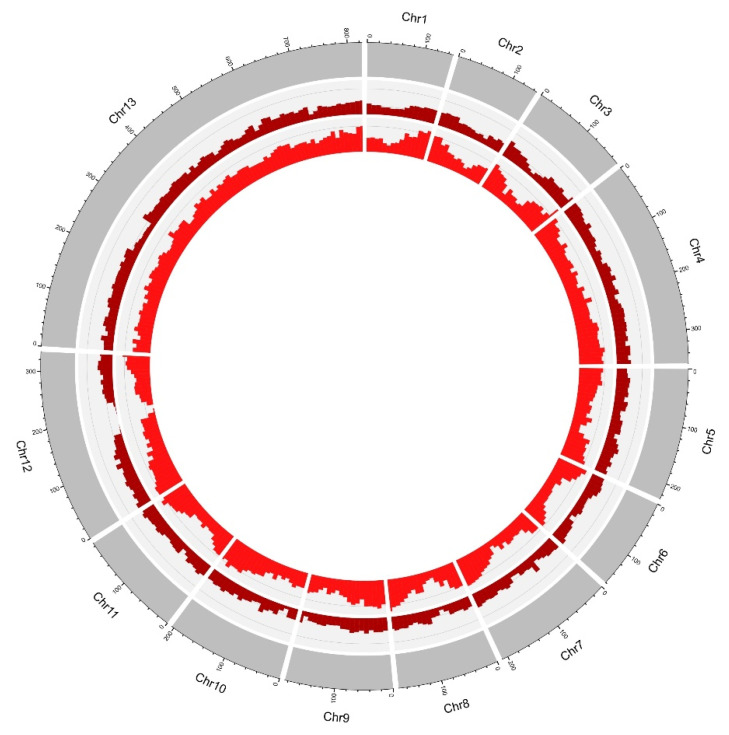
Distribution of meadow and mountainous ecotypes’ SNPs in contrast to reference genome assembly. From outer to inner circles: chromosomes, meadow ecotype’s SNPs, and mountainous ecotype’s SNPs.

To perform a basic comparison of the different ecotypes’ genomes, two additional samples, one representing the meadow, and another the mountainous ecotype, were sequenced. Illumina PE150 sequencing yielded 364 and 370 M reads for the meadow and mountainous ecotype individuals, respectively. However, the usability of such a short-read data set was limited and does not allow detailed comparative analyses of genomes characterized by very high proportions of repetitive elements. Nonetheless, we were able to calculate pairwise distances between the constructed genome assembly and the additional samples based on the total number of detected SNPs (Figure 6) and analyze their distribution across the genomes (Figure 7). Additionally, the SNP abundances within genes and on the genome level were compared and expressed as the average distance between neighboring SNPs. The results showed that the mountainous ecotype was the most diverged one, while a substantially higher density of SNPs was detected within genes compared to the entire genome.

## 3. Discussion

Here, we present a draft genome assembly for *Chouardia litardierei*, a non-model monocot species from the Hyacinthaceae family. By combining long-read sequencing and the chromosome conformation capture method, we successfully assembled a high-quality 3.7 Gbp genome of *C. litardierei*, and the obtained result agrees with the previously reported genome size for the species [8]. By inspecting the Taxonomy Browser of the NCBI repository (https://www.ncbi.nlm.nih.gov/data-hub/taxonomy/tree/?taxon=4447 (accessed on 17 April 2023)), it became obvious that, within monocots, most species with assembled genomes are either of substantial economic importance (maize, wheat, rice, pineapple, banana, asparagus, jams, onion, garlic, etc.) or their wild relatives. In a lower taxonomic rank, within the order Asparagales, assembled genomes of well-known groups of orchids (i.e., *Dendrobium*, *Vanilla*, and *Phalaenopsis*) and *Asparagus* prevail, once again showing bias towards species of economic importance. Of less closely related species to *C. litardierei* within Asparagales that have available genome assemblies, few can be mentioned. The genome assembly of *Asparagus setaceus* was 720 Mb in size and characterized by 1393 scaffolds and a 2.19 Mb N50 scaffold value [22]. The 1.19 Gb *Dendrobium nobile* genome assembly reached a 64.5 Mb N50 scaffold value [23], while the *Cymbidium goeringii* genome, of very similar size to the genome of *C. litardierei* (3.99 vs. 3.70 Gbp, respectively), had an N50 scaffold size of 178.2 Mb [24]. Since we reached the N50 scaffold value of more than 210 Mb, this indicates the high contiguity of the assembled genome. In addition, the BUSCO score of over 97% additionally supported this conclusion. Additionally, a revealed chromosome size distribution perfectly matches the only known karyotype for this species reported by Siljak-Yakovlev et al. [7].

The annotation of repetitive elements revealed that TEs occupy almost 70% of the genome, with LTR retrotransposons being the most abundant class. Such a result was not surprising, as it is well known that genome size in plants greatly depends on these elements’ abundance [25,26]. Our results are mostly consistent with those reported for other monocot species. For instance, the *Hordeum vulgare* ssp. *vulgare* genome (5.1 Gbp in size, Poaceae) consists of 72.8% TE elements [27], the genome of *Areca catechu* (2.6 Gbp, Arecaceae) of 80.4% [28], and that of *Allium fistulosum* (Amaryllidaceae 11.2 Gbp) of 69% [29]. At the same time, genomes of some other monocots, such as *Setaria italica* (423 Mbp, Poaceae) [30], *Trichopus zeylanicus* (713 Mbp, Dioscoreaceae) [31], and *Kobresia myosuroides* (400 Mbp, Cyperaceae) [32] reportedly harbor substantially fewer transposable elements, occupying 41%, 36%, and 44.9% of their genomes, respectively. As mentioned, since the abundance of TEs strongly influences the genome size, species characterized by smaller genomes usually have fewer TEs as well.

To reach high accuracy for the genome annotation, we implemented various approaches to annotate protein-coding genes. Out of the 27,257 predicted genes, most of them (85.8%) were matched with a functional annotation in at least one public database, while almost half of them (44.5%) were matched in all selected databases. 

The genus *Prospero* represents a closely related group to *C. litardierei*. It formerly belonged to *Scilla*, and the same is true for the *Chouardia* studied here. *Prospero*, especially the *P. autumnale* s.l. group, is well known for its structural genome rearrangements and multiple ploidy levels and was used as the model group for research on the evolutionary implications of karyotype differentiation [33,34]. In addition, Siljak-Yakovlev et al. [7] hypothesized that the genome of *C. litardierei* could have originated through whole-genome duplication events. To verify whether the *C. litardierei* genome shares some characteristics with *P. autumnale* s.l., or has indeed originated through a whole-genome duplication event, we performed intra-genome syntenic gene block analysis. However, no clues supporting any of these assumptions were found, as it became clear that the *C. litardierei* genome did not undergo any such structural rearrangements since only a few gene blocks co-occurred on more than one position across the genome. In contrast to the limited distribution area of *C. litardierei*, *P. autumnale* s.l. stretches across the Mediterranean basin, so we can assume that the vast distances and subsequent geographical isolation eventually led to the establishment of groups of populations characterized by specific cytotypes. 

The evolutionary analysis confirmed the positioning of *C. litardierei* and the entire Hyacinthaceae family within Asparagales. At the same time, it confirmed that the genus *Asparagus*, the closest relative to *C. litardierei* with the available draft genome, can hardly be treated as a close relative since the divergence time was estimated at around 50 Mya. This result further emphasizes the importance of our work, as *C. litardierei* is an obvious representative of, so far, a neglected phylogenetic group in terms of available genomic resources. Regarding other phylogenetic relationships and divergence times among the analyzed representatives of various monocot groups, our results were in high agreement with other similar studies [32,35,36].

Comparative analyses of the assembled genome and two individuals belonging to different ecotypes were of limited success. A shotgun-sequencing approach with a 150 bp read length greatly limited our abilities for in-depth analyses. Nonetheless, we were able to extract SNPs and analyze their distribution across the genomes. The results supported our initial assumption that a higher degree of relatedness is present between the seashore and meadow ecotypes, while the mountainous ecotype is more diverged and possibly represents a separate lineage. In addition, the analysis of SNP distribution within and outside protein-coding regions indicated an above-average density of variations within the coding regions. This result shows that some regions are evolving at a higher pace than others, possibly as a consequence of yet undetermined selective pressures. However, such a conclusion based on only three individuals is likely premature, as research that would include a substantially larger sample set is required for more reliable conclusions. The reasoning behind performing this analysis was to determine if there are any indications of ongoing divergence processes among the lineages, which in the end, we successfully identified.

## 4. Materials and Methods

### 4.1. Sample Collection, DNA Extraction, and Sequencing

Fresh leaf material from an individual belonging to the seashore ecotype of the studied species was collected and immediately placed in a silica gel for rapid desiccation. High-molecular-weight DNA extraction following the CTAB method [37], DNA quality control, PacBio HiFi, and Hi-C library preparation and sequencing were performed by Brigham Young University DNA Sequencing Center (Provo, UT, USA). In short, PacBio circular consensus sequencing (CCS) libraries were constructed and sequenced on the 8M SMRT cell of the PacBio Sequel II instrument (Pacific Biosciences of California, Menlo Park, CA, USA), while Hi-C libraries were constructed using a Dovetail^®^ Omni-C^®^ Kit and sequenced on an Illumina HiSeq platform (Illumina Inc., San Diego, CA, USA) to generate 2 × 250 paired-ends reads.

### 4.2. Genome Assembly

Before the assembly process, the genome size of *C. litardierei* was estimated using a *k*-mer counting method and the tool Jellyfish 2.3.0 [38]. PacBio HiFi reads were processed by Jellyfish to determine their *k*-mer distribution, and the *k*-mer size of 19 was selected. The genome size was estimated as the total number of counted *k*-mers divided by the highest frequency of *k*-mers that occurred. PacBio HiFi reads were assembled into contigs using hifiasm 0.16.1-r375 [39]. Racon 1.4.17 [40] was used in an attempt to improve read quality before the assembly process. The contigs obtained by hifiasm were polished using two rounds of consensus correction with Racon and PacBio HiFi reads.

The generated contigs were scaffolded into pseudochromosomes using Hi-C data. Hi-C reads were first processed following the Omni-C data analysis and quality control protocol, recording valid ligation events and removing PCR duplicates. After initial processing, the Hi-C reads were mapped to contigs using the Juicer tool [41], producing contact map information. To detect misjoins in contigs and to join contigs located on the same chromosomes, 3D-DNA v180922 [42] was used. For the manual rearrangement of obtained scaffolds into pseudochromosomes, we used the Juicebox tool [43]. The same software was also used to generate a FASTA file with sequences corresponding to 13 manually assembled chromosomes, with Ns filling the gaps between scaffolds within each chromosome. This final assembly was further polished with PacBio HiFi reads using the HyPo polisher [44]. HiFi reads were mapped to the final assembly using the minimap2 tool 2.23 [45] with the option “-x map-hifi”.

The initial and the final assemblies’ quality was assessed using Quast [46] and BUSCO 5.2.2 [47] to compare the assembly to the gene content of Viridiplantae_odb10 “https://busco-archive.ezlab.org/frame_plants.html (accessed on 7 December 2022)”. For the genome assembly visualization, we used shinyCircos [48]. The GC content of the assembled genome was calculated using an in-house script. The density of total repeats, DNA transposons, *Copia* repeats, and *Gypsy* repeats was determined from the data obtained through the repetitive element annotation, as explained in the next subsection. Intra-genomic syntenic analysis was performed using SyMAP 5.4.0 [49] with the default parameters.

### 4.3. Repetitive Elements Annotations

First, the known repeat sequences of Viridiplantae were identified based on Dfam [50] hidden Markov Model (HMM) sequence profiles (release 3.6) using RepeatMasker 4.1.2-p1 [51] and the NCBI/RMBLAST search engine. Furthermore, the de novo repeat identification approach was implemented using RepeatModeler2 2.0.2 [52] with Tandem Repeats Finder 4.10 [53], RECON 1.0.8 [54], and RepeatScout 1.0.6 [55] which enabled LTR Structural analysis. RepeatClassifier (a module of RepeatModeler2) was implemented for further classification of de novo repeats into unknown and classified classes. All three groups of repeats were used in a combined masking step to construct the finally masked version of the genome. The final BUSCO analysis against Viridiplantae_odb10 was performed on this version of the masked genome.

### 4.4. RNA Isolation and Sequencing

For support of the gene prediction, RNA-Seq data were generated. Total RNA was extracted from roots, leaves, flowers, and unripe fruit using a Monarch^®^ Total RNA Miniprep Kit (New England BioLabs, Ipswich, MA, USA). The manufacturer’s protocol, with an on-column DNAse digestion step, was followed. Eluted RNA was quantified utilizing spectrometry, and integrity was verified by Agilent Bioanalyzer 2100 electrophoresis using an RNA 6000 Nano Kit (Agilent Technologies, Santa Clara, CO, USA). RNA was stored at −80 °C until processing.

RNA sequencing was performed using the Ion Proton system. Total RNA was enriched for the poly-A mRNA fraction using a Dynabeads^®^ mRNA DIRECT™ Micro Kit (Thermo Fisher Scientific, Waltham, MA, USA). The isolated mRNAs were used for RNA-Seq library preparation using the procedure for low-input RNA from the Ion Total RNA-Seq kit v2 (Thermo Fisher Scientific, Waltham, MA, USA). The RNA was fragmented using RNase III enzymatic digestion followed by ligation of Ion Adapters using four different barcodes to retain tissue specificity. The samples were reverse transcribed, purified, and cDNA amplified, and the obtained library was verified using the High Sensitivity DNA Kit (Agilent Technologies, Santa Clara, CO, USA). The libraries, in equimolar amounts, were pooled together and amplified by emulsion PCR using an Ion OneTouch™ 2 System and Ion PI Hi-Q OT2 200 Kit. Template-positive particles were enriched using Dynabeads^®^ MyOne™ Streptavidin C1 beads (Thermo Fisher Scientific, Waltham, MA, USA) on an Ion OneTouch™ ES system. The obtained enriched particle samples were sequenced on PI™ Chip v3 using the Ion PI™ Hi-Q™ Sequencing 200 Kit (Thermo Fisher Scientific, Waltham, MA, USA) following the manufacturer’s protocol. The quality check of trimmed reads after processing was performed by the FastQC tool [56].

### 4.5. Gene Prediction and Annotation

To predict protein-coding sequences, we used several approaches implemented using different tools. First, gene models were developed with the MAKER genome annotation pipeline (MPI 3.01.04) [57] incorporating: (1) RNA-seq data, (2) protein-based evidence based on 139,388 Asparagales clade proteins downloaded from the NCBI RefSeq database “https://www.ncbi.nlm.nih.gov/refseq/ (accessed on 9 January 2023)”, and (3) ab initio gene predictions obtained using SNAP 2006-07-28 [58] and Augustus 3.2.3 [59]. For SNAP software training, MAKER models with a max AED threshold of 0.25 and a minimum length of 50 amino acids were used, and for training Augustus, the BUSCO pipeline was employed following the method of Card et al. [60]. Three runs of MAKER were run iteratively to obtain most gene models with an AED score above 0.5.

Additional ab initio gene prediction was obtained using GeneMark-ES [61], followed by de novo and genome-guided transcriptome assembling using the Trinity 2.14.0 software [62] (default parameters). For the construction of the genome-guided transcriptome, the GMAP tool [63], and SAMtools 1.14 [64] were used to map the reads to the previously constructed genome assembly and to obtain a coordinate sorted bam file, respectively. The transcriptomes obtained by Trinity were used as inputs for the PASA alignment assembly pipeline 2.5.2 [65] (default parameters). The obtained transcriptome was further used to identify and extract likely coding regions using PASA’s Transdecoder software. For homology-based gene prediction, the Asparagales protein set was used again. The proteins were mapped to the previously constructed genome using the miniprot tool [66].

Finally, the MAKER gene annotations together with the PASA transcriptome, PASA likely coding regions, protein alignments obtained by miniprot, and ab initio predictions obtained by GeneMark-ES, were analyzed using EVidenceModeler 2.0.0 [67], producing the final consensus gene set.

Recognized protein-coding genes were functionally annotated based on entries in the NCBI NR database [68], Swiss-Prot [69], InterPro [70], and EggNOG [71] databases, using BLASTP searches with an E-value cut-off of 1.0 × 10^−5^. For the visualization of the obtained results, a Venn diagram was constructed.

### 4.6. Genome Evolution Analysis

Orthologous groups were identified using OrthoFinder 2.5.4 [72] and protein sequences from *Ananas comosus* (L.) Merr., *Arabidopsis thaliana* (L.) Heynh., *Asparagus officinalis* L., *Dioscorea rotundata* Poir., *Hordeum vulgare* L., *Musa acuminata* L., *Oryza sativa* L., and *Zea mays* L. Single-copy ortho-groups were collected and aligned using MUSCLE 3.8.1551 [73]. The alignments were concatenated into a super-alignment and filtered using Gblocks 0.91.1 [74]. The phylogenetic trees were constructed using RaxML-NG 0.9.0 [75].

Divergence time estimation was performed using the MCMCTree tool in the PAML 4.9j package [76]. Analyses were run using default settings (200,000 generations with a burn-in of 2000 iterations). The calibration points for the *O. sativa*–*H. vulgare* (42–62 Mya), *A. comosus*–*M. acuminata* (103–117 Mya), and *D. rotundata*–*A. thaliana* (142–164 Mya) were obtained from the TimeTree database [77] “http://www.timetree.org (accessed on 6 April 2023)”. Finally, for the identification of gene families’ expansions and contractions, CAFE5 [78] was implemented.

### 4.7. Intra-Species Comparison of the Genomes

In addition, to perform a basic comparative analysis of genomes from different ecotypes, two individuals, each from a distinct ecotype (meadow and mountainous ecotypes, Samples 1 and 2, respectively), were sampled. DNA was extracted from dried leaf material using the GenElute™ Plant Genomic DNA Miniprep Kit (Sigma–Aldrich, St. Louis, MO, USA) and sent to Novogene (UK) Company Limited for short-fragment libraries preparation and PE150 sequencing on an Illumina NovaSeq platform (Illumina Inc., San Diego, CA, USA). The paired-end reads were mapped to the constructed genome assembly using the BWA tool 0.7.17 [79], and the variants were called using the FreeBayes tool [80,81]. The obtained data were used to assess the pairwise genetic distances between analyzed individuals belonging to different ecotypes. In addition, the abundance of the SNPs within protein-coding regions was analyzed using an in-house script.

## Figures and Tables

**Table 1:** Summary results for the final assembly of the *Chouardia litardierei* genome.

Sequence	
Assembly size (bp)	3,698,590,323
GC content (%)	42.90
Number of scaffolds	9916
Number of scaffolds (≥50 kbp)	1803
Longest scaffold (bp)	824,692,949
Scaffold N50 size (bp)	210,067,440
Number of contigs	3111
Number of contigs (≥50 kbp)	1611
Longest contig (bp)	54,979,118
Contig N50 size (bp)	12,914,002
Pseudochromosome	
Number	13
Size range (Mbp)	145.64–824.69
BUSCO score	
Complete BUSCOs (%)	97.4
Complete and single-copy BUSCOs (%)	89.9
Complete and duplicated BUSCOs (%)	7.5
Fragmented BUSCOs (%)	2.4
Missing BUSCOs (%)	0.2

**Table 2:** Classification of the repetitive elements in the *Chouardia litardierei* genome.

	Percent (%)	Total Length (Mbp)
Retrotransposons		
LINE	2.99	110.72
SINE	0.06	2.14
LTR	63.25	2339.37
DNA Transposons	3.67	135.60
Unclassified	7.81	288.98
Satellites	0.14	5.10
Simple repeats	1.42	52.63
Low complexity	0.31	11.53
Rolling circles	0.58	21.30
Small RNA	0.70	25.88
Total	80.90	2991.99

**Table 3:** RNA sequencing data from different *Chouardia litardierei* tissues.

	Root	Leaf	Flower	Developing Fruit
No. of raw reads	22,769,326	24,504,881	28,584,130	23,731,351
Total nucleotides [Mbp]	3013.5	3360.2	3918.0	3070.6
GC content [%]	47.90	49.18	49.65	51.26
Average length [bp]	123.0	137.1	137.1	129.4
Min-max length [bp]	8–383	8–381	8–381	8–384
No. of reads after trimming	22,079,991	23,884,368	27,738,059	23,053,022
Total nucleotides after trimming [Mbp]	2957.7	3309.0	3855.2	3018.6
Average read length after trimming [bp]	134.0	138.5	139.0	131.0

**Table 4:** Summary of the gene prediction and annotation results of *Chouardia litardierei*.

Gene Prediction	
Number of predicted genes	27,257
Number of predicted genes in 13 pseudochromosomes	23,297
Chr1	1237
Chr2	1152
Chr3	1477
Chr4	2137
Chr5	1757
Chr6	1309
Chr7	1429
Chr8	1513
Chr9	1435
Chr10	1589
Chr11	1344
Chr12	2373
Chr13	4545
Mean gene length (bp)	3109.9
Mean CDS length (bp)	764.1
Mean exon length (bp)	181.0
Mean intron length (bp)	728.0
Avg. exons per gene	4.2
Gene annotation	
NCBI NR annotated (%)	17,602
EggNog annotated (%)	14,691
InterPro annotated (%)	22,633
Swiss-Prot annotated (%)	12,782
Number of annotated genes	23,398
Proportion of annotated genes (%)	85.8%

## Data Availability

All the obtained data (PacBio, Hi-C, RNA-Seq, and WGS reads), as well as the final genome assembly and predicted gene models, are available in the NCBI database under the BioProject ID PRJNA974736.
